# Long-term *in vivo* biodistribution and toxicity study of functionalized near-infrared persistent luminescence nanoparticles

**DOI:** 10.1038/s41598-018-29019-z

**Published:** 2018-07-13

**Authors:** Xia Sun, Junpeng Shi, Xiaoyan Fu, Yi Yang, Hongwu Zhang

**Affiliations:** 1 Key Lab of Urban Pollutant Conversion, Institute of Urban Environment, Chinese Academy of Sciences, Xiamen, 361021 China; 2 University of Chinese Academy of Sciences, Beijing, 100049 China; 3 College of Materials Science and Engineering, Xiamen University of Technology, Xiamen, 361024 China

## Abstract

Near-infrared (NIR) persistent luminescence nanoparticles (NPLNPs) have become one of the most promising candidates for bioimaging. Different from the other fluorescence nanoprobes, the NIR persistent luminescence of NPLNPs can last for a long time after excitation, double exposure that is nanoparticles and light exist during the long-term bioimaging. However, to date, the potential risk of nanoparticles and NIR persistent luminescence of NPLNPs is still unknown. In this study, Cr3 + -doped zinc gallate, Zn_1.1_Ga_1.8_Sn_0.1_O_4_:Cr^3+^ (ZGO), the most promising NPLNPs in bioimaging, was chosen as a representative for potential risk assessment. We evaluated the potential risk of nanoparticles and NIR persistent luminescence of ZGO for a long period of time. *In vitro* study showed that the ZGO possessed a low cytotoxicity. *In vivo* biodistribution results showed that the ZGO mainly accumulated in the reticuloendothelial system after intravenous injection and could be gradually cleared from the body by digestive system. In addition, the ZGO did not exhibit appreciable toxicity in mice over a period of 60 days. It’s also worth mentioning that long-term NIR persistent luminescence of ZGO did not exhibit obvious toxicities both *in vitro* and *in vivo*. Our results provide important information with regards to the risk of NPLNPs in long-term bioimaging.

## Introduction

In the past few years, optical imaging has received much attention in biomedicine due to its advantages of high sensitivity, non-invasiveness, no radioactivity, and low cost^1,2^. The effectiveness of optical imaging heavily depends on the properties of the optical imaging probes. With the development of nanoscience and nanotechnology, a series of optical imaging nanoprobes possessing excellent properties, such as metal nanoclusters^3^, semiconductor quantum dots (QDs)^4^, up-conversion nanoparticles (UCNPs)^5,6^, near-infrared (NIR) persistent luminescence nanoparticles (NPLNPs)^7^, have been synthesized and applied to optical imaging. Among these nanoprobes, NPLNPs are considered to be one of the most promising types of candidates for bioimaging. The NIR luminescence of NPLNPs can last for several hours (even several days) after the excitation is ceased, and thus, imaging can be achieved without requiring an *in situ* excitation light source, which completely avoided the background noise from *in situ* excitation and dramatically increased the signal-to-noise ratio (SNR) as well as times of bioimaging^8–11^.

To date, quite a few NPLNPs have been reported for *in vitro* and *in vivo* imaging. NPLNPs based on a silicate matrix as the first generation bioimaging nanoprobes were applied for *in vivo* imaging for the first time^12^. Richard *et al*. synthesized the NPLNPs CaMgSi_2_O_6_:Eu^2+^/Mn^2+^/Pr^3+^ using a sol-gel method, which were excited by UV radiation. These PLNPs displayed a strong NIR persistent luminescence and allowed real-time and high SNR *in vivo* imaging^13^. We also synthesized high quality NPLNPs of SiO_2_/CaMgSi_2_O_6_:Eu^2+^/Pr^3+^/Mn^2+^ using a simple template method, which have a uniform spherical morphology and tunable sizes. The *in vivo* real-time distribution of the NPLNPs can be detected more than 1 hour after injection into the abdomen of a mouse^14^. However, the first generation of NPLNPs based on a silicate matrix was faced with some drawbacks, such as the short persistent luminescence time (<3 h) and UV excitation, which seriously hinder their further application in bioimaging. With the development of NIR persistent luminescence materials, the photostimulated NIR persistent phosphor Cr^3+^-doped LiGa_5_O_8_ was synthesized by a solid-state reaction method^15^. The phosphor exhibited a long NIR persistent luminescence of more than 1,000 h after excitation by a UV lamp, which has largely solved the drawback of the short persistent luminescence time of the first generation NPLNPs. Using a sol-gel method, LiGa_5_O_8_:Cr^3+^ nanoparticles were synthesized and further applied to ultrasensitive, deep-tissue, and long-term image tracking of cells *in vivo*^16^. However, this generation of NPLNPs still need UV excitation, and thus, it cannot fundamentally solve the drawback of the SNR decrease with the decay of NIR persistent luminescence for *in vivo* imaging.

In 2012, Pan *et al*. reported a revolutionary Cr^3+^-doped zinc gallate persistent phosphor of Zn_3_Ga_2_Ge_2_O_10_:Cr^3+^ that exhibited strong NIR emission at 696 nm with a super-long persistent luminescence of more than 360 h. More importantly, the new generation phosphors can be rapidly, effectively and repeatedly excited by visible light^17^, which solved the drawback of the first two generations of NPLNPs. Since then, NIR persistent luminescence imaging has entered a period of rapid development. Currently, many NPLNPs based on Cr^3+^-doped zinc gallate have been synthesized and applied to *in vitro* and *in vivo* imaging. Scherman *et al*. reported Cr^3+^-doped zinc gallate NPLNPs (ZnGa_1.995_O_4_:Cr^3+^), whose NIR persistent luminescence can be excited *in vivo* through living tissues using highly penetrating low energy red LEDs. After surface functionalization with polyethylene glycol (PEG), these NPLNPs can be used for tumor targeting and cell tracing *in vivo*^18^. Moreover, triple-doped zinc gallogermanate NPLNPs (Zn_1.25_Ga_1.5_Ge_0.25_O_4_:Cr^3+^/Yb^3+^/Er^3+^) were synthesized by a hydrothermal method in combination with calcination for a short time. Using *in situ* red LED excitation, Yan *et al*. first realized long-term and high SNR tumor targeting imaging through the oral administration NPLNPs^19^. We also developed a series of Cr^3+^-doped zinc gallate NPLNPs, including Zn_1.1_Ga_1.8_Ge_0.1_O_4_:Cr^3+^/Eu^3+^@SiO_2_^20^, ZnGa_2_O_4_:Cr^3+^/Eu^3+ ^^21^, ZnGa_2_O_4_:Cr^3+^@HMS^22^, and ZnGa_2_O_4_:Cr^3+^/Sn^4+ ^^23^ for tumor targeting imaging, drug delivery, and deep tissue renewable imaging *in vivo*. In addition, Cr^3+^-doped zinc gallate NPLNPs have also been applied to other fields, such as biosensing^24^, multimodal imaging^25^ and photodynamic therapy^26^.

Despite the encouraging results of using Cr^3+^-doped zinc gallate NPLNPs in bioimaging, there remain many unresolved problems with respect to the understanding of their potential risk *in vitro* and *in vivo* in long-term bioimaging. In addition, different from the other optical imaging probes (QDs and UCNPs), the NIR persistent luminescence of NPLNPs can last for long time after excitation. Although the long-term NIR persistent luminescence is very beneficial to the high SNR bioimaging, whether the long-term NIR persistent luminescence in bioimaging can cause phototoxicity is still unknown. These potential risk studies of NPLNPs are of the utmost importance for their future biomedical application; Though a few works has been done to evaluated nanoparticles toxicity in cells^27^, more potential risk study of Cr^3+^-doped zinc gallate NPLNPs relative to nanoparticles and NIR persistent luminescence is still need to be done. In the present study, we synthesized PEG modified Cr^3+^-doped zinc gallate NPLNPs, Zn_1.1_Ga_1.8_Sn_0.1_O_4_:Cr^3+^ (ZGO). We systematically evaluated the *in vitro* risk of nanoparticles and NIR persistent luminescence of ZGO in three different cell lines through a series of cellular assays, and then studied the long-term *in vivo* biodistribution through persistent luminescence imaging. Finally, to determine their potential *in vivo* risk, we carried out a long-term toxicology analysis of ZGO in mice over a period of 60 days.

## Results and Discussions

### ZGO synthesis and functionalization

**Figure 1:**
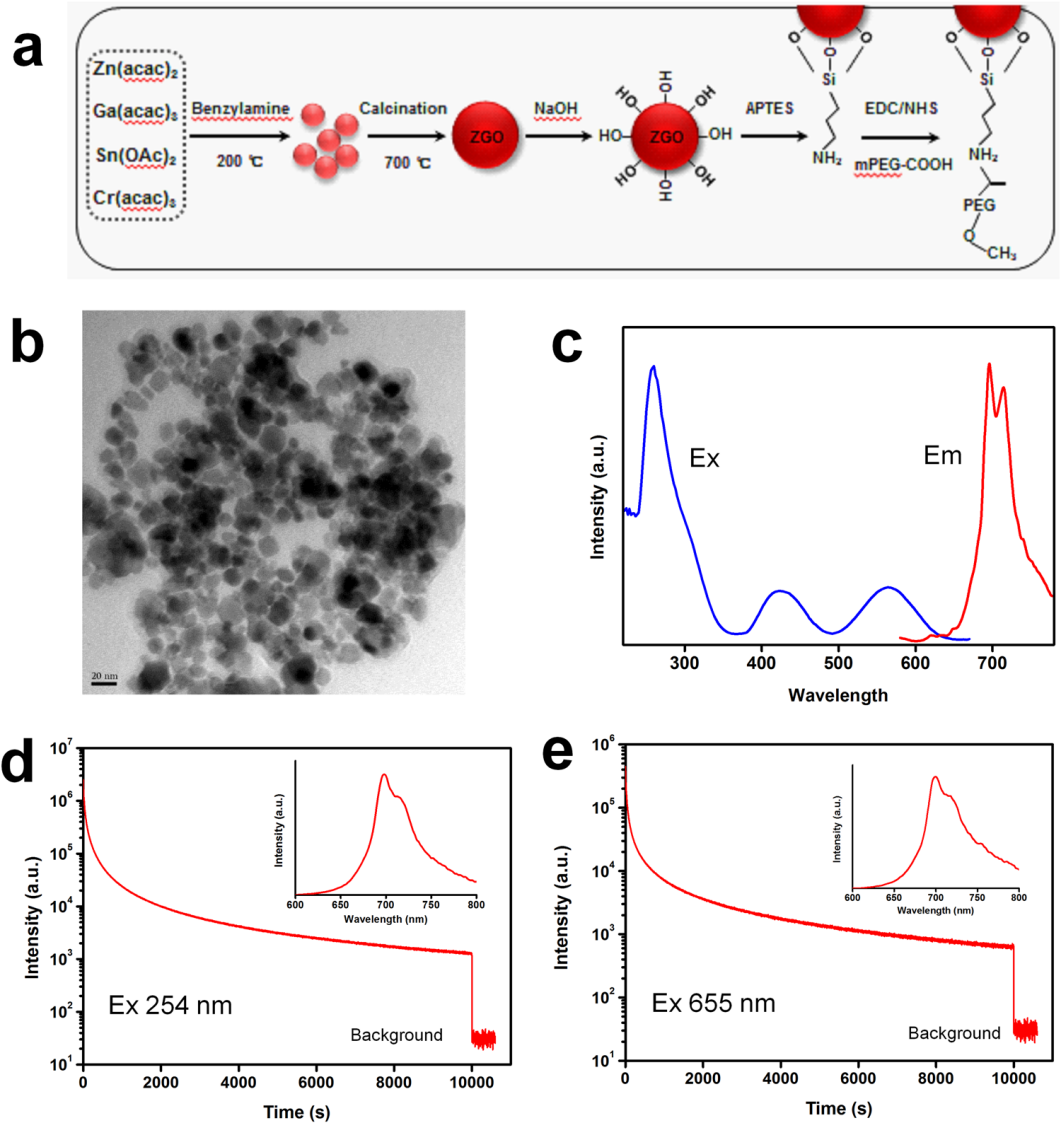
(**a**) Scheme for the synthesis and surface functionalization of ZGO, (**b**) transmission electron micrograph (TEM) images of ZGO, (**c**) excitation (em 700 nm) and emission (ex 254 nm) spectra of ZGO, and (**d**,**e**) NIR persistent luminescence decay curve of ZGO at 700 nm after 5 min of irradiation with a 254 nm UV lamp and 655 nm LED; insets: the afterglow emission spectra after irradiation with a 254 nm UV lamp and 655 nm LED.

The synthesis and the surface functionalization of Cr^3+^-doped zinc gallate NPLNPs, Zn_1.1_Ga_1.8_Sn_0.1_O_4_:Cr_0.005_ (ZGO) is shown in Fig. 1a. We first synthesized the precursor substance of ZGO using a solvothermal method at 200 °C. Then, the ZGO was obtained through low temperature calcination at 700 °C for a short time. Original ZGO possess poor biocompatibility and solubility, which limit the further application in bioimaging. Grafting ZGO with PEG is effective at increasing the biocompatibility and solubility. Thus, in order to improve the biocompatibility and solubility, the ZGO was PEGylated through three different reactions. TEM images show that the as-prepared ZGO particles are monodispersed with an average diameter of 20–30 nm (Fig. 1b). The XRD pattern of ZGO shows the spinel phase of ZnGa_2_O_4_ (JCPDS 71-0843) (Additional file 1: Fig. S1), which indicated that the ZGO was successfully synthesized. The emission and excitation spectra of ZGO are shown in Fig. 1c. We can find a narrow-band emission peak at 700 nm under excitation at 254 nm. The excitation spectrum shows a broad band with three main peaks at 260, 416, and 576 nm monitored at 700 nm. In addition, the ZGO exhibits excellent NIR persistent luminescence properties. As shown in Fig. 1d, the ZGO has a strong NIR emission at 700 nm (Fig. 1d inset), and its NIR persistent luminescence shows a slow decay and persisted for more than 3 h after excitation with 254 nm light for 5 min. More importantly, the ZGO also exhibits a strong NIR emission (Fig. 1e inset) and slow persistent luminescence decay (å 3 h) after excitation with a 655 nm LED for 5 min (Fig. 1e), suggesting that the ZGO can be effectively excited through the deep tissue by the high penetrability of the 655 nm LED. In addition, the successful synthesis of PEG-functionalized ZGO was demonstrated by Fourier-transform infrared (FTIR) spectroscopy (Additional file 1:Fig. S2).

### *In vitro* cytotoxicity study of ZGO

**Figure 2:**
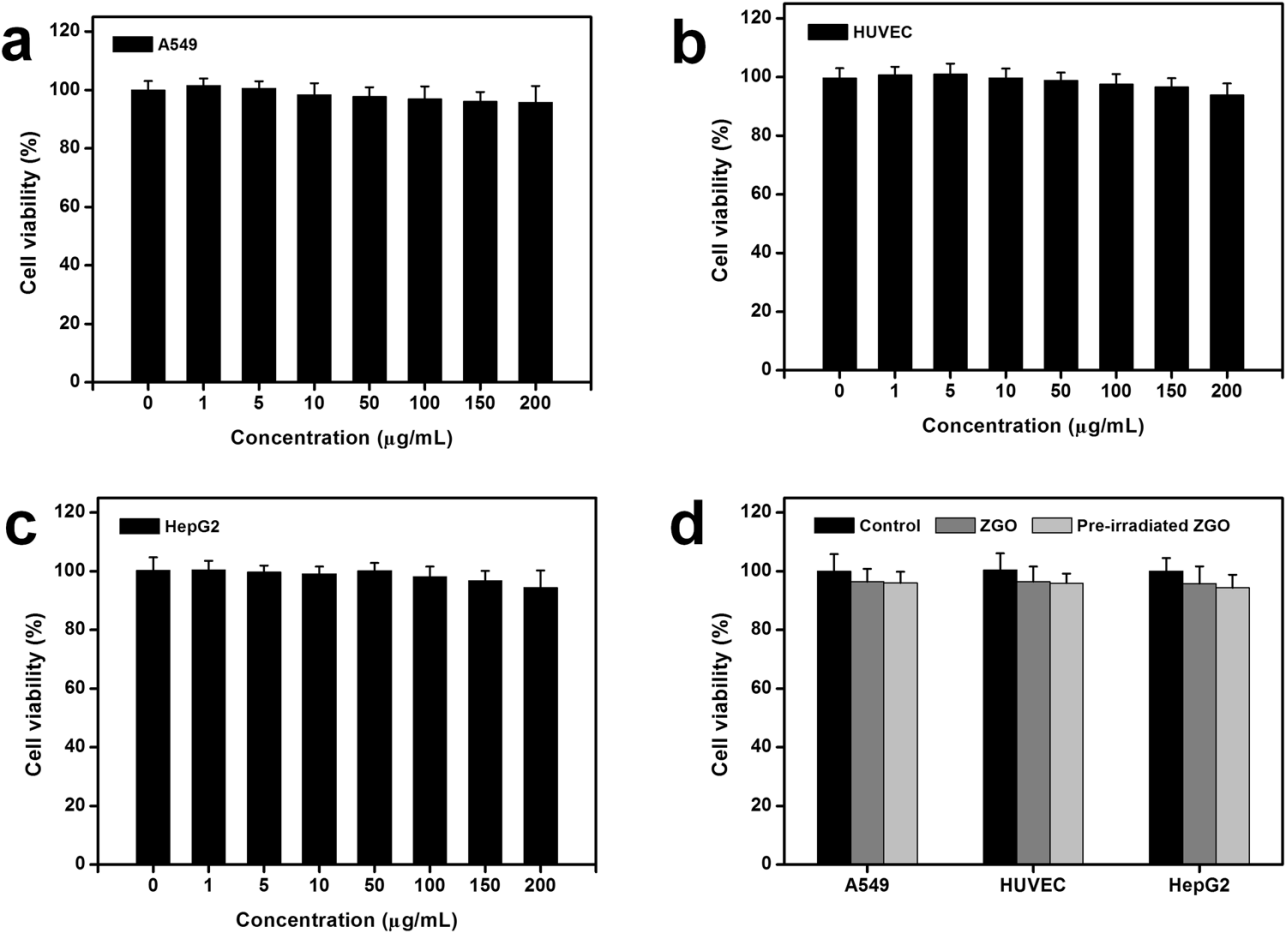
Cytotoxicity of ZGO. (**a**–**c**) MTT assay of the A549 cell line, HUVEC cell line, and HepG2 cell line under different concentrations, and (**d**) MTT assay of the three cell lines with ZGO or pre irradiated ZGO. The cells were incubated with ZGO or pre-irradiated ZGO for 24 h. Data represent the mean ± SD (n = 6). Pre-irradiated ZGO means that the ZGO was pre-irradiated with a 254 nm UV lamp for 5 min before experiment.

We carried out a series of studies to evaluate the cytotoxicity of ZGO. Three different cells, human lung carcinoma cells (A549), human hepatocellular carcinoma cells (HepG2) and human umbilical vein endothelial cells (HUVEC), were chosen as model cells because of their high probability of exposure to nanoparticles *in vivo*^28,29^. After exposure to ZGO in cells for 24 h, slight fluorescence signals were found by confocal microscopy imaging, it means that a small part of ZGO were uptaken by cells (Additional file 1:Fig. S3). Cell viability, which is a fundamental method for assessing the impact of a toxicant on cell health, based on the standard MTT method was then performed among these different cell lines. Figure 2a–c showed the relative cell viabilities of A549, HUVEC and HepG2 cells after 24 h of exposure to different concentrations of ZGO. No statistically significant differences of cell viability were observed in the absence or presence of ZGO for the three different cell lines. As shown in Fig. 2a, over 95% of A549 cells retained normal viability even when the ZGO concentration went up to 200 μg/mL. Similar phenomena were also found in the other two types of cells (HUVEC and HepG2) (Fig. 2b,c). Moreover, previous studies have suggested that some nanoparticles such as nano-TiO_2_ tend to exhibit higher phototoxicity when they were irradiated with UV photoirradiation; they have limited cytotoxicity under a normal situation and show higher cytotoxicity, even leading to significant cell damage, under UV photoirradiation^30–33^. The NIR persistent luminescence of ZGO can last for long time after excitation by the UV photoirradiation or a 655 nm LED lamp. To determine whether the NIR persistent luminescence of ZGO causes phototoxicity and damages the cells or not when ZGO was excited before exposure to cells, cell viabilities under this situation were also evaluated. As shown in Fig. 2d, compared with the controls, no significant differences in cell viability were observed when ZGO was excited before exposure to the three cell lines, suggesting the safety of the NIR persistent luminescence of ZGO. All of these results indicate that the ZGO has little effect on cell viability.

**Figure 3:**
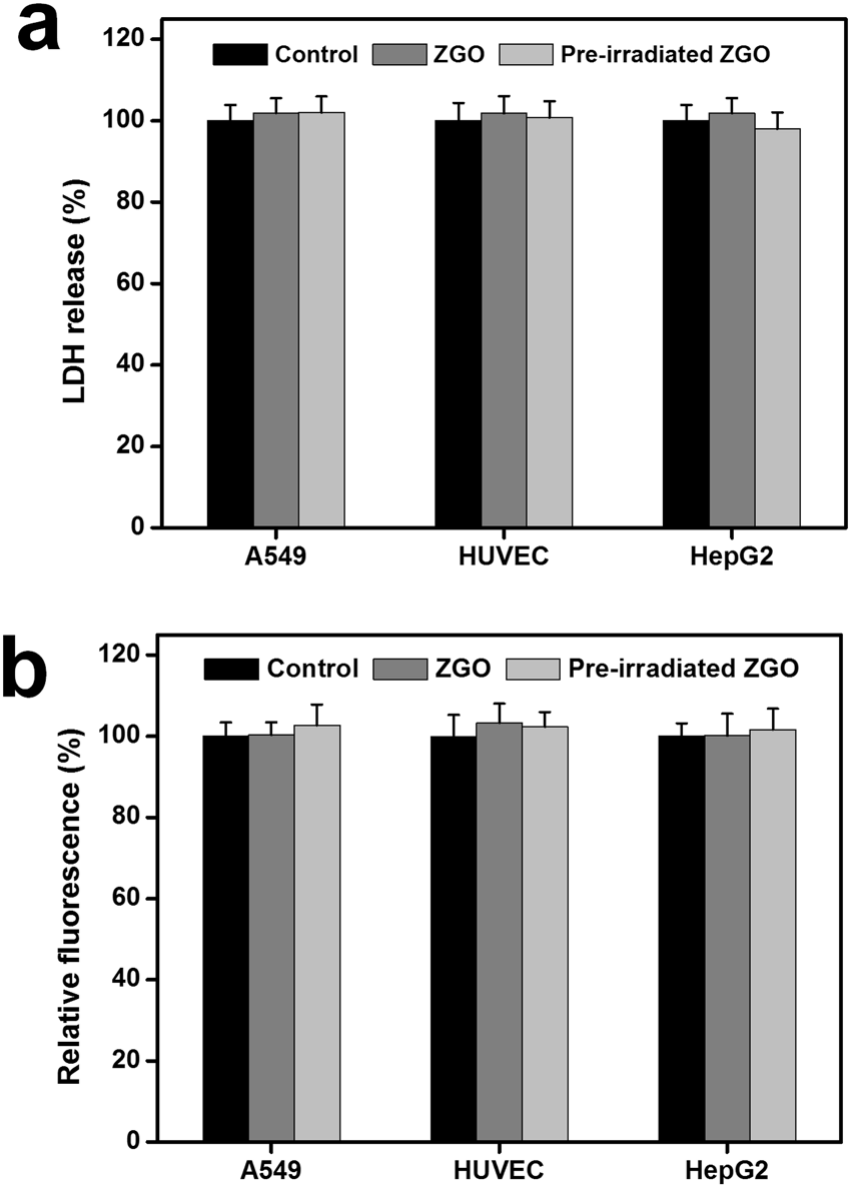
(**a**) Relative LDH level and (**b**) relative ROS level. The cells were incubated with ZGO and pre- irradiated ZGO for 24 h. The data represent the mean ± SD (n = 6).

In view of the significance of cell membrane integrity in cell-nanoparticle interactions, we then carried out a further cytotoxicity investigation of ZGO on three cell lines by a LDH assay. Here, we chose a high exposure concentration (200 μg/mL) of ZGO for the follow-up experiment based on the MTT results. Meanwhile, the effect of the NIR persistent luminescence of ZGO on cell membranes was still examined. As shown in Fig. 3a, no significant differences in LDH release were observed compared with the control after exposure to ZGO in three different cell lines. Similarly, we also did not find significant differences in the LDH release in the group with UV lamp pre-irradiation. These results indicate that the ZGO, whether pre-irradiated by a UV lamp or not, did not damage the cell membrane.

Furthermore, we measured the oxidative stress of three different cell lines after exposure to ZGO. The results showed that A549, HUVEC and HepG2 cells treated with ZGO at the concentration of 200 μg/mL for 24 h produced a negligible amount of ROS, which had no significant differences in comparison to that of the control (Fig. 3b). Similarly, compared with the control group, pre-irradiated ZGO by a UV lamp also did not induce a noticeable increase of intracellular ROS generation. These results suggest the low oxidative stress-related cytotoxicity of ZGO. Consistent with our results, Ramirez-Garcia *et al*. found that PEGylated ZnGa_1.995_Cr_0.005_O_4_ wither pre-excitation or not have no effect on the production of ROS in human breast cancer cells^27^.

**Figure 4:**
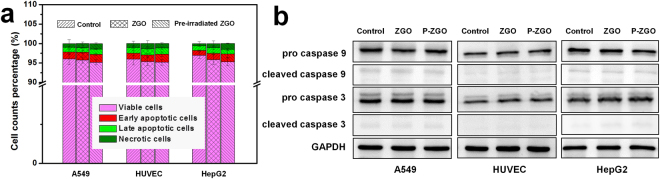
Cell apoptosis detection of ZGO. (**a**) Cell apoptosis assay using Annexin V/PI staining (n = 3). (**b**) Protein levels of caspase-3 and caspase 9. GAPDH served as a loading control. Full gel and blots are presented in Supplementary information. The cells were incubated with ZGO or pre-irradiated ZGO (P-ZGO) for 24 h. Results are expressed as the mean ± SEM (n = 3).

We finally evaluated the apoptosis of the three cell lines exposed to ZGO using Annexin V-FITC and PI staining. As shown in Fig. 4a, no appreciable increase of cell apoptosis and no significant cell death were seen in the three different cell lines after incubation with ZGO in both cases (whether ZGO was excited or not). To confirm this finding, the protein levels of caspase 9 and caspase 3 were detected by western blot analysis. The activation of caspase-9 initiates the programmed cell apoptosis, while the activation of caspase-3 is a key event during the late stage of apoptosis^34^. Compared with controls, the two protein expression levels showed relatively few changes after exposure to ZGO in both cases, and non-cleavage of important caspases was observed (Fig. 4b). Taken together, all of the results above reveal that ZGO has negligible toxicity.

### Long-term *in vivo* imaging and biodistribution

**Figure 5:**
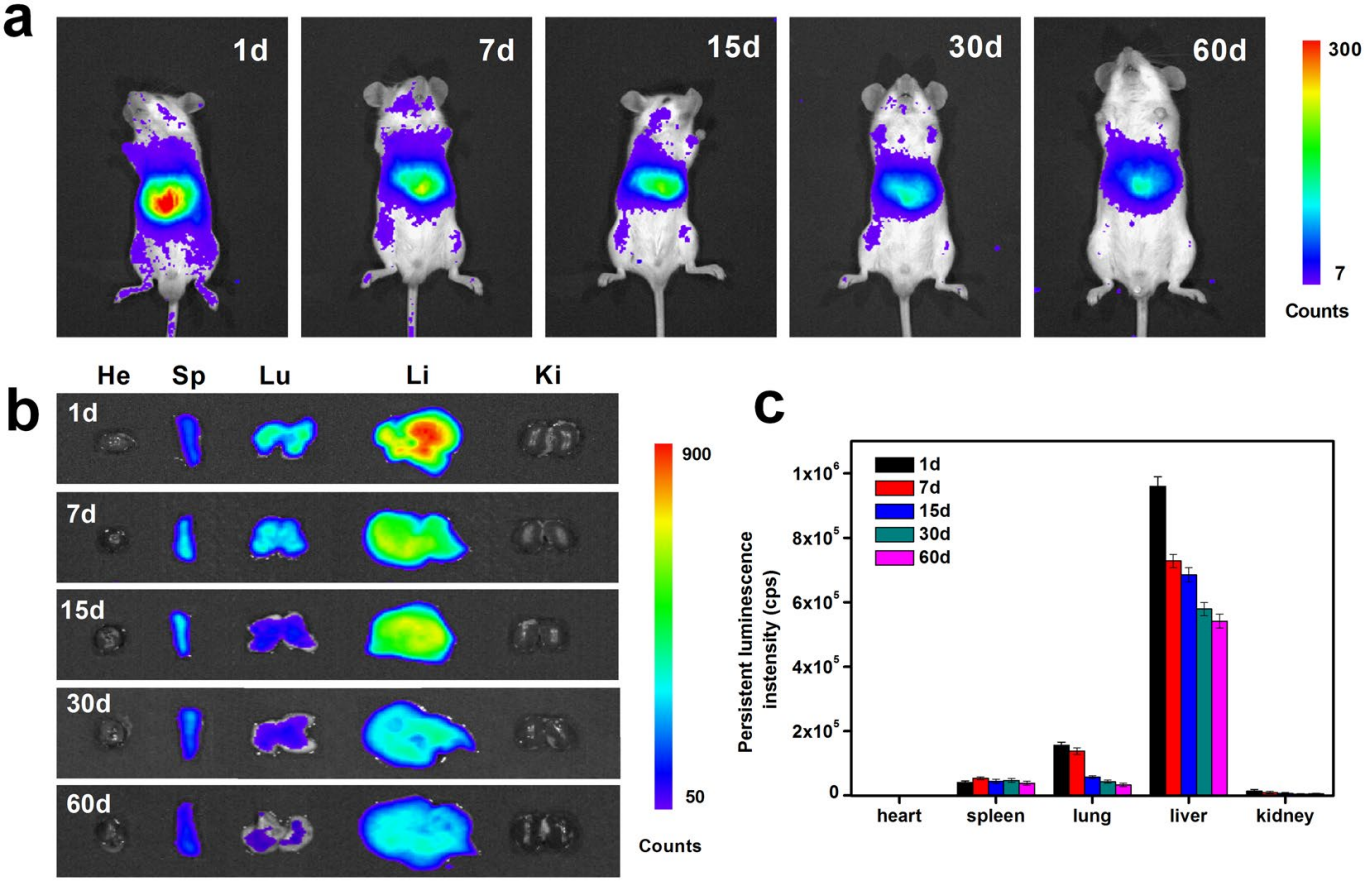
*In vivo* and *ex vivo* persistent luminescence imaging. (**a**) *In vivo* persistent luminescence images of Balb/c mice with the intravenous injection of ZGO (10 mg/kg) at different time points, (**b**) *ex vivo* persistent luminescence imaging of the heart (He), liver (Li), spleen (Sp), lungs (Lu) and kidneys (Ki) collected from Balb/c mice injected with ZGO at different time points, and (**c**) persistent luminescence intensities of ZGO from isolated organs at different time points. The data represent the mean ± SD (n = 5)

For long-term *in vivo* imaging studies, Balb/c mice were intravenously injected with 10 mg/kg ZGO. At different time points, the mice were anesthetized, *in situ* irradiated by a 655 nm LED for 5 min and imaged using an IVIS Lumina II imaging system. As shown in Fig. 5a, a persistent luminescence signal was observed in the most of the body at one day post-injection, and the signal was predominantly in the positions of reticuloendothelial system (RES) organs. As time progressed, the persistent luminescence signal in the body and at the positions of RES organs gradually decreased. At 60 days post-injection, the persistent luminescence signal still can be observed at the positions of RES organs, but the luminescence signal is obviously lower than at previous time points (Fig. 5a). The results suggest that ZGO is distributed throughout the body through blood circulation and accumulates in several major organs after injection. The ZGO would stay inside the bodies of the mice for a long period of time and then is slowly excreted from the body through systemic circulation over time.

To further evaluate the long-term biodistribution of ZGO in mice, major organs were collected at different time points after injection, irradiated by a 655 nm LED for 3 min and then imaged by an IVIS Lumina II imaging system. Persistent luminescence images of various organs are shown in Fig. 5b. At 1 day post-injection, the strong persistent luminescence signals were detected in the liver, spleen and lungs, and no obvious persistent luminescence was detectable from the heart and kidney. The persistent luminescence signals in the liver and lungs gradually decreased as time went on (Fig. 5c). The signals remained detectable for up to 60 days in the liver, spleen and lungs, indicating that ZGO remains inside the mice bodies long-term. Consistent with the *in vivo* imaging, *ex vivo* images confirmed that the ZGO uptake and retention took place primarily in the liver, spleen and lungs. As we all know, the reticuloendothelial system (RES), a network of cells and tissues throughout the body generally found in the connective tissue, liver, spleen and lungs, has a high capacity to metabolize and degenerate a multitude of xenobiotics^35,36^. The high-level accumulation of ZGO in organs such as the liver, spleen and lungs suggested that the rapid uptake of the ZGO was intercepted by macrophages in the RES, and then, it may be partially metabolized causing the loss of the persistent luminescence signals over time. Such an uptake mechanism involving RES is the classical behavior of nanoparticles *in vivo*^37^. For example, up-converting nanoparticles of various types are usually deposited mainly in the liver, spleen and lungs, where they can persist for a long time^29^. It is noteworthy that lower persistent luminescence signals were also detected in the stomach and intestine as time progressed (Additional file 1: Fig. S4), which suggested that the clearance of ZGO in the RES may be dependent mainly on the digestive system via hepatobiliary transport. A similar clearance pathway has been reported in Ag_2_S quantum dots and NaYF_4_:Yb,Tm up-conversion nanoparticles^38,39^.

### *In vivo* toxicology study of ZGO

**Figure 6:**
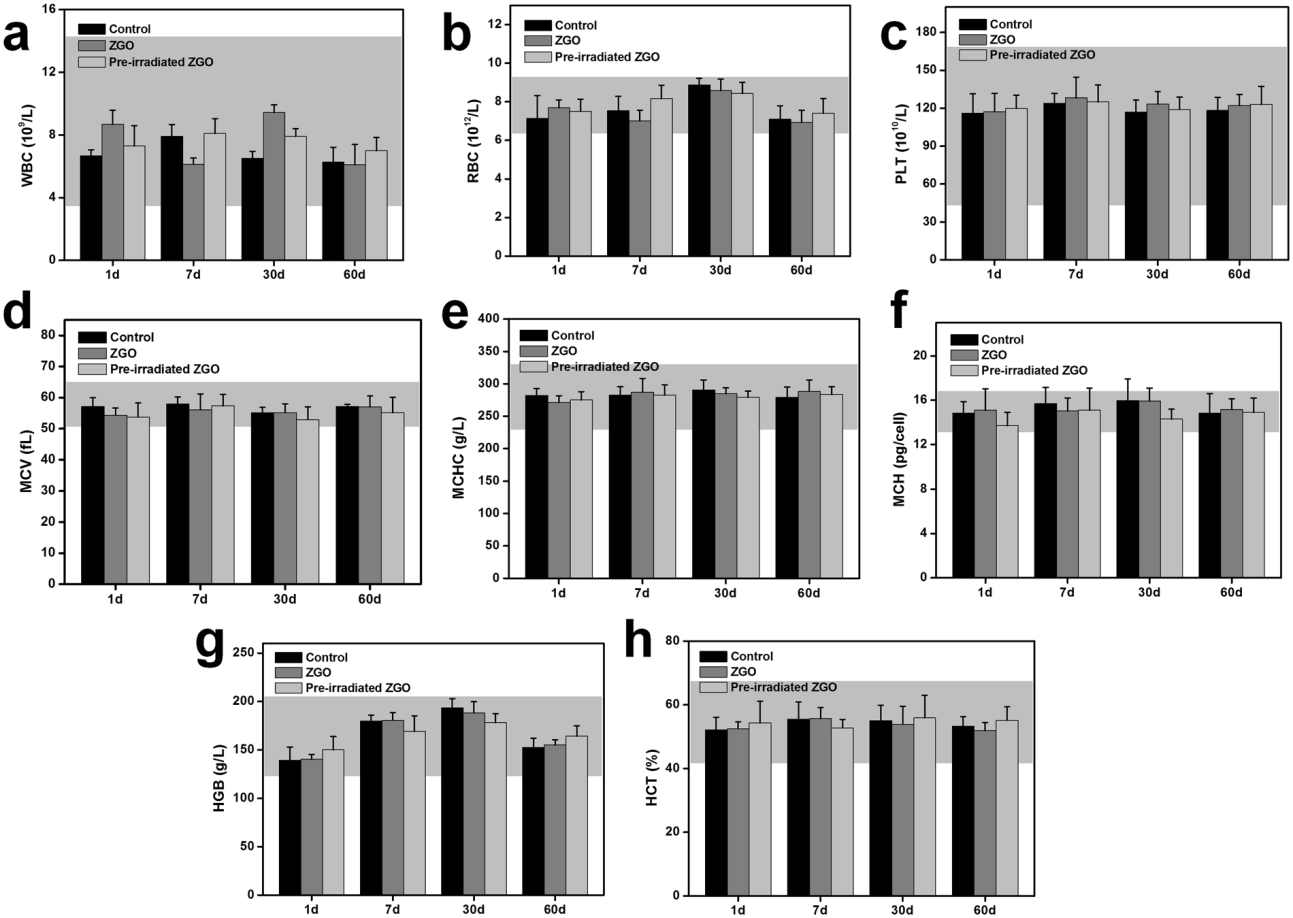
Blood hematology data of Balb/c mice treated with ZGO or pre-irradiated ZGO with a 254 nm UV lamp at 1, 7, 30 and 60 days. (**a**) White blood cells (WBC), (**b**) red blood cells (RBC), (**c**) platelet count (PLT), (**d**) mean corpuscular volume (MCV), (**e**) mean corpuscular hemoglobin (MCH), (**f**) mean corpuscular concentration (MCHC), (**g**) hemoglobin (HGB), and (**h**) hematocrit (HCT) levels in the blood at various time points were tested. All blood hematology data were within the normal range, and the data are based on five mice per group. Gray areas in the figures show the normal reference ranges of hematology data for healthy male Balb/c mice.

As ZGO could be retained in the bodies of mice for a long period of time after injection, a more systematic study was then carried out to look for the *in vivo* potential long-term toxicity. Healthy Balb/c mice were intravenously injected with ZGO at a dose of 10 mg/kg and closely monitored for 60 days. ZGO pre-irradiated with a 254 nm UV lamp was also set as one of the factors to evaluate the possible effect of NIR persistent luminescence of ZGO *in vivo*. No signs of apparent weakness, spontaneous animal death and significant body weight gain or loss were observed within 60 days (Additional file 1: Fig. S5). Nanoparticles are particulate materials with a small size and large surface area, potentially leading to an immune response and the ability to alter related hematological factors once they get into the body^39–41^. Therefore, serum biochemistry and a complete blood panel assay were used to more quantitatively evaluate the potential toxicity of ZGO on the mice. Blood was collected at 1, 7, 30, and 60 days after the intravenous injection of ZGO. For routine blood examination, the white blood cells (WBC), red blood cells (RBC), platelet count (PLT), mean corpuscular volume (MCV), mean corpuscular hemoglobin (MCH), mean corpuscular concentration (MCHC), hemoglobin (HGB), and hematocrit (HCT) were measured, as shown in Fig. 6. It was found that all of the parameters were in the normal reference range^42^, and in the tested group, these parameters show no obvious difference compared with the control group during the monitoring period. It indicated that the numbers and shapes of white blood cells, red blood cells and platelets were at a normal level and did not indicate a significant risk associated with ZGO treatment, and so did as NIR persistent luminescence of ZGO.

**Figure 7:**
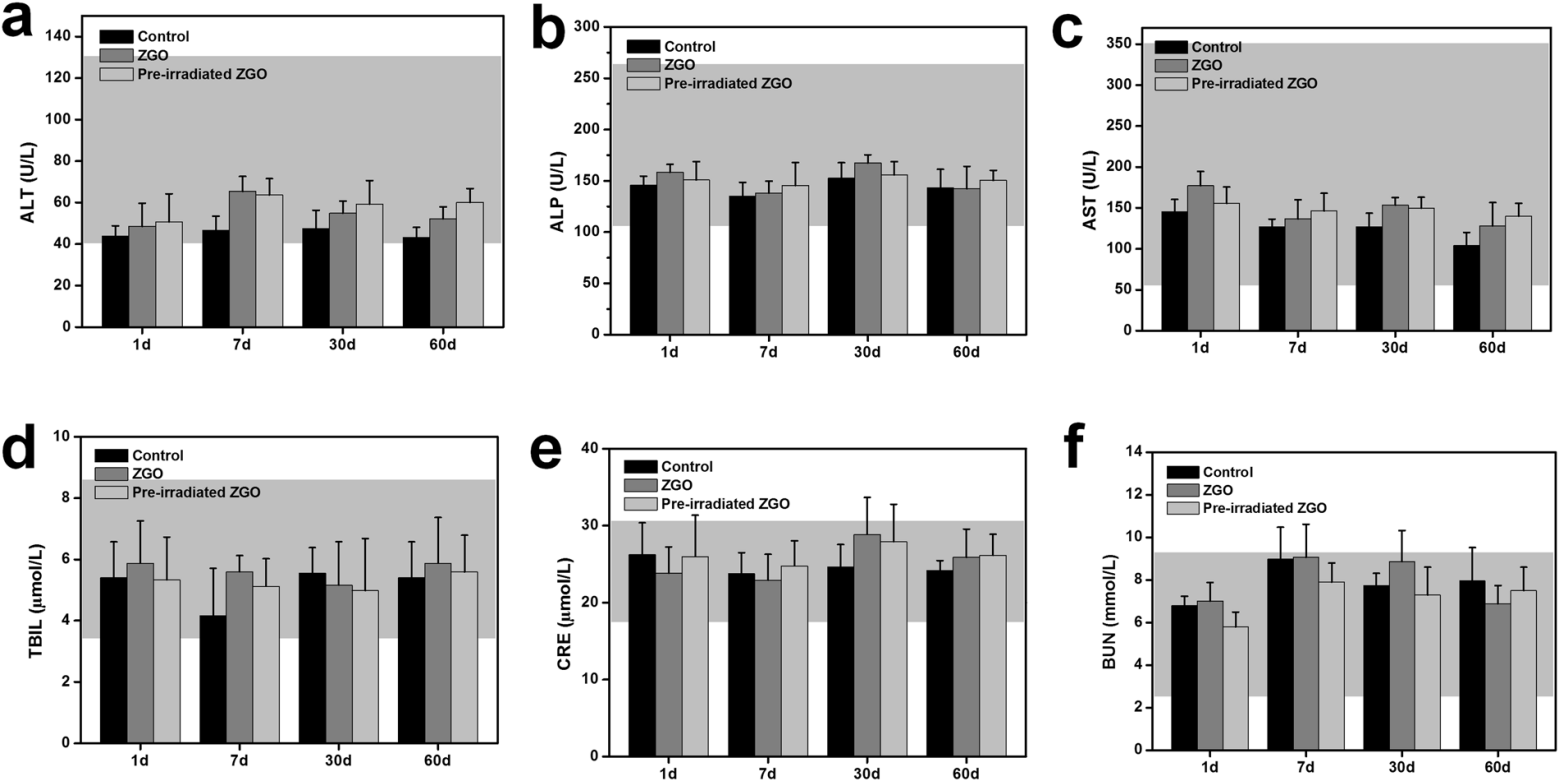
The serum biochemical analysis of Balb/c mice treated with ZGO or pre-irradiated ZGO with a 254 nm UV lamp at 1, 7, 30 and 60 days. (**a**) Alanine aminotransferase (ALT), (**b**) alkaline phosphatase (ALP), (**c**) aspartate aminotransferase (AST), (**d**) total bilirubin, (**e**) blood urea nitrogen (BUN), and (**f**) creatinine (CRE) levels in the blood at various time points were tested. All serum biochemical data were within the normal range, and the data is based on five mice per group. Gray areas in the figures show the normal reference ranges of hematology data for healthy male Balb/c mice.

In the hematological analysis, various serum biochemical parameters were measured with particular attention paid to liver and kidney function. As shown in Fig. 7, the liver function indicators including the alkaline total bilirubin (TBIL), phosphatase (ALP), alanine aminotransferase (ALT), and aspartate aminotransferase (AST) in the ZGO-treated groups at different times post-injection were at similar levels as those for the control mice. Indicators of kidney function, including creatinine (CRE) and urea nitrogen (BUN), were also within normal ranges and were similar to these of control mice. These results show no obvious injury to the liver and kidney with ZGO exposure in mice (whether it was pre-irradiated or not), even at long exposure times.

**Figure 8:**
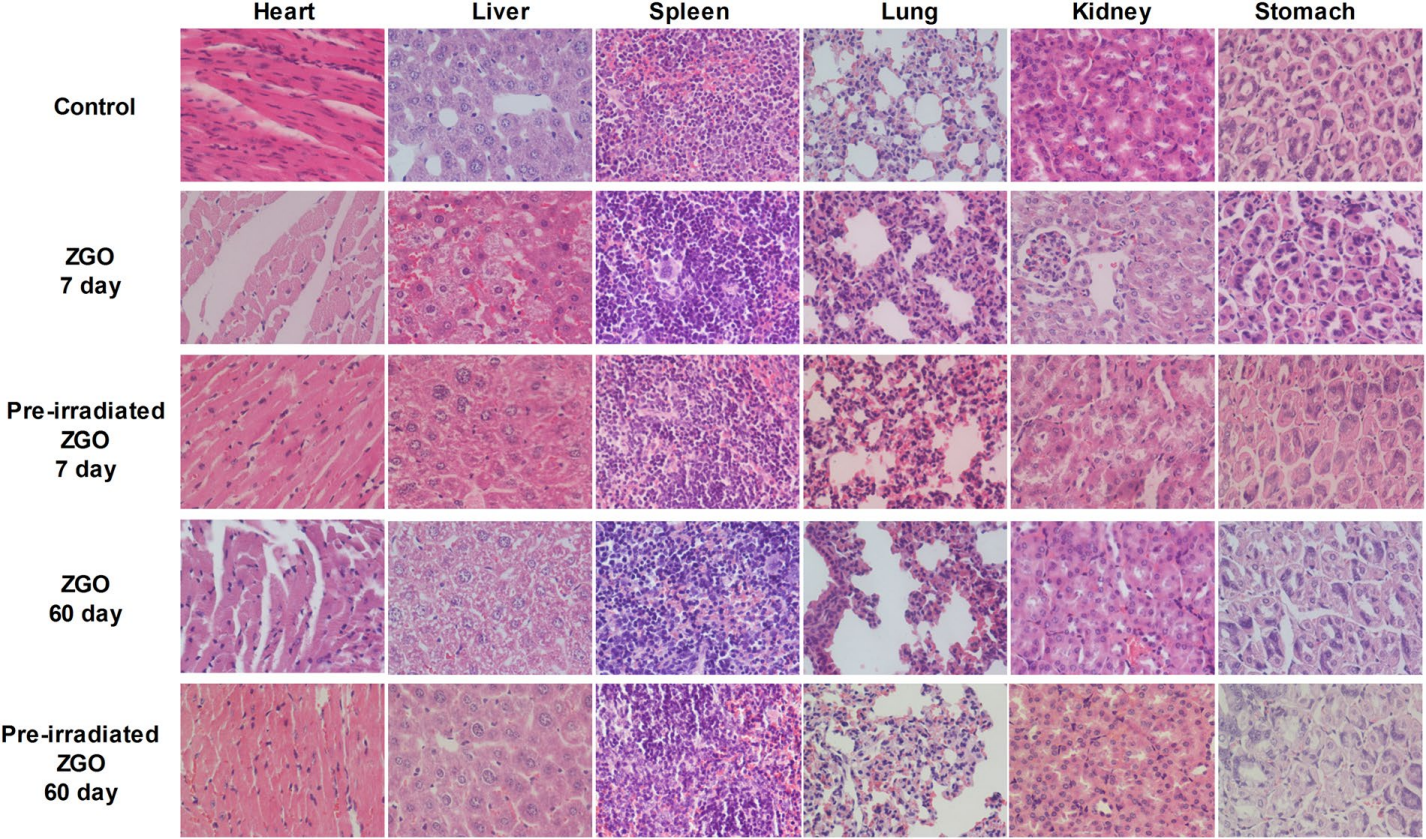
Hematoxylin and eosin stained images of major organs including the heart, liver, spleen, lungs, kidneys and stomach from the mice injected with ZGO or pre-irradiated ZGO with a 254 nm UV lamp at 7, and 60 days post-injection.

Based on long-term *in vivo* biodistribution studies, the major organs from mice were sliced for H&E staining and histological examination to determine whether or not ZGO exposure caused tissue damage, inflammation or lesions. The structures of the organs from ZGO and pre-irradiated ZGO exposed mice at different times points exhibited hardly any difference from the control group (Fig. 8). No apparent histopathological abnormalities or lesions were found in any of the experimental groups. All of these data suggested that no significant toxicity was induced by ZGO injection (whether it was pre-irradiated or not), not only short-term but also long-term, even up to 60 days.

## Conclusion

In summary, we have evaluated the potential risk of nanoparticles and NIR persistent luminescence of ZGO during the long-term bioimaging. *In vitro* cytotoxicity studies showed that ZGO did not induce obvious changes in the cell viabilities, the injury of cell membranes, oxidative stress and apoptosis in three different cell lines. Furthermore, the NIR persistent luminescence imaging of Balb/c mice and isolated organs indicated that the ZGO mainly accumulated in the liver, spleen and lungs after intravenous injection and was cleared from the body by the digestive system. More importantly, the ZGO did not cause significant toxicities in mice for 60 days, as indicated by blood biochemistry, hematological and histological analyses. Besides, it is worth mentioning that NIR persistent luminescence of ZGO did not cause obvious toxicities both *in vitro* and *in vivo* as well. Although more careful toxicology studies are necessary, the lack of obvious toxicity shown in our study encourages future development of Cr^3+^-doped zinc gallate NPLNPs for *in vivo* biomedical research.

## Methods

### Materials

Zn(acac)_2_·xH_2_O, Ga(acac)_3_, Cr(acac)_3_, and Sn(CH_3_COO)_2_ were purchased from Sigma Aldrich (Sigma-Aldrich, USA). Benzylamine, dimethylformamide (DMF), (3-aminopropyl) triethoxysilane (APTES), N-(3-dimethylaminopropyl)-N′-ethylcarbodiimide hydrochloride (EDC) and N-hydroxysuccinimide (NHS) were purchased from Aladdin (China). Human lung carcinoma cells (A549) and human hepatocellular carcinoma cells (HepG2) were purchased from the cell resource center of the Shanghai Institutes for Biological Sciences (SIBS, China). Human umbilical vein endothelial cells (HUVEC) were purchased from Key-gen Biotech (China). Other biological materials such as fetal bovine serum (FBS), culture medium and supplements were purchased from Life Technologies (USA).

### Synthesis of ZGO

The ZGO was synthesized by a solvothermal method in combination with subsequent calcination in air. Zn(acac)_3_·xH_2_O, Ga(acac)_3_, Sn(CH_3_COO)_2_ and Cr(acac)_3_ were mixed with 30 mL of benzylamine according to the following chemical formula: Zn_1.1_Ga_1.8_Sn_0.1_O_4_:Cr_0.005_. The reaction mixture was transferred into a Teflon-lined stainless steel autoclave and heated to 200 °C for 48 h. Then, the precipitate was centrifuged and washed with ethanol and water. The precipitate was dried at 60 °C and finally calcined at 700 °C for 2 h in air.

### Surface functionalization of ZGO

The surface functionalization of the ZGO was carried out according to a previous method^9^. In brief, the ZGO was dispersed in a NaOH solution (5 mM) by sonication for 1 h and then stirred for 24 h at room temperature. The hydroxylated ZGO was collected by centrifugation and dried at 50 °C in a vacuum oven. One hundred milligrams of hydroxylated ZGO was dispersed in 100 mL of DMF under sonication for 30 min. Then, 300 μL of APTES was added under stirring. The above solution was continuously stirred for 48 h at room temperature. After that, the aminated ZGO was collected by centrifugation and washed with ethanol and water several times.

To obtain PEGylated ZGO, 50 mg of aminated ZGO and 200 mg of methoxy poly(ethylene glycol) carboxyl acid (mPEG_5000_-COOH) was dispersed in 50 mL of PBS (pH = 7.4) under sonication for 30 min. Subsequently, 50 mg of EDC and 100 mg of NHS were added to the above solution under stirring. The mixture was continuously stirred for 24 h at room temperature. The precipitates were collected by centrifugation and washed with water and ethanol several times. The final products were placed in a vacuum oven for 24 h at 50 °C.

### Characterizations

Transmission electron microscopy (TEM) images were obtained on a HITACHI H-7650 microscope. The X-ray diffraction (XRD) pattern was recorded on a PANalytical X’pert Pro diffractometer. The photoluminescence spectra and NIR persistent luminescence spectra were recorded on an FLS920 fluorescence spectrometer. The FTIR spectra were recorded on a Nicolet iS10 spectrometer. The *in vivo* imaging experiments were performed on an IVIS Lumina II imaging system.

### Uptake of ZGO by cells

The uptake of ZGO by cells was analyzed by confocal microscopy imaging. Cells were seeded in 35 mm culture dishes and treated with ZGO 50 μg/mL for 24 h. Then, the medium was removed and the cells were washed with PBS on a shaker to remove the redundant ZGO. Finally, the cells were imaged by the LSM 710 Laser Scanning Confocal Microscope (Zeiss, Jena, Germany).

### Cell culture

All culture media were supplemented with 10% FBS and 1% penicillin-streptomycin solution. A549 cells were grown in Ham’s F-12K (Kaighn’s) medium with 2.5 g/L sodium bicarbonate; HUVEC cells were grown in Ham’s F-12K (Kaighn’s) medium with 0.03–0.05 mg/ml endothelial cell growth supplements (ECGS) and 0.1 mg/L heparin; and HepG2 cells were grown in minimum essential media with 1.5 g/L sodium bicarbonate and 0.11 g/L sodium pyruvate. All cells were cultured at 37 °C in a humidified atmosphere containing 5% CO_2_. Considering the possible effect of the persistent luminescence of ZGO on cells, the ZGO pre-irradiated with a 254 nm UV lamp for 5 min was added to the cell experiment as one of the factors.

### Cell viability assay

The MTT assay was carried out to examine the cell viability of ZGO. Cells were seeded onto 96-well plates and treated with ZGO at various concentrations. After 24 h of treatment, 0.5 mg/mL 3-(4,5-dimethyl-2-thiazolyl)-2,5-diphenyl-2-H-tetrazolium bromide (MTT, Sigma-Aldrich) was added to each well and incubated for 4 h at 37 °C. The resulting formazan crystals were solubilized by DMSO, and the absorbance was measured using a microplate reader (Molecular Devices, USA).

### LDH release assay

The effect of ZGO on the cell membrane was measured by lactate dehydrogenase (LDH) release using a Cytotoxicity Detection Kit (Roche, Germany). Briefly, cells were seeded onto 96-well plates. After 24 h of treatment with ZGO, the medium was removed and transferred into corresponding wells of another 96-well plate. Cells were washed with PBS and lysed by Triton-X (1%). Finally, reaction mixtures were added and incubated for 30 min at 25 °C. The absorbance of the samples were measured at 492 nm using a microplate reader.

### Measurement of the ROS

The reactive oxygen species (ROS) were measured using a peroxide-sensitive fluorescent probe, 2,7-dichlorofluorescein diacetate (DCFH-DA, Sigma-Aldrich). Briefly, cells were seeded in 6-well plates and treated with ZGO for 24 h. Then, the cells were washed with PBS and incubated with 10 µM DCFH-DA for 30 min at 37 °C. After that, the cells were washed and resuspended in ice-cold PBS. Finally, the fluorescence intensities of the samples were measured with a microplate reader.

### Cell apoptosis assay

The Annexin V-FLUOS Staining Kit (Roche) was used to detect cellular apoptosis. Briefly, after 24 h of treatment with ZGO, cells were collected and washed with PBS. Then, the cells were resuspended in the Annexin-V-Fluos labeling solution containing the Annexin V-Fluos labeling reagent and PI at room temperature for 15 min. Finally, the cells were analyzed using a flow cytometer (Beckman Coulter, USA).

### Western blot analysis

Western blot analysis was carried out as previously described^43^. Cells lysates were prepared with RIPA buffer (Beyotime Inst. Biotech, China) containing protease inhibitor cocktail (Merck, Germany). The protein concentration was assayed with a BCA protein assay kit (Thermo Scientific, USA). Equal amounts of protein for different samples were subject to sodium dodecyl sulfate polyacrylamide gel electrophoresis (SDS-PAGE) and then transferred to the PVDF membranes. The membrane was blocked with 5% nonfat dry milk in TBST for 1 h at room temperature and incubated with primary antibodies at 4 °C overnight. Thereafter, the blots were incubated with 1:2000 IgG-HRP secondary antibody (Abcam Trading Company Ltd., Shanghai, China) for 1 h at room temperature. Finally, the antibody-reactive bands were revealed by enzyme-catalyzed chemiluminescence (ECL, Thermo Fisher) and were quantified by densitometry analysis using Image Pro Plus version 6.0 software. Antibodies were against GAPDH (1:1000, abcam), caspase-3 (1:1000, abcam), and caspase-9 (1:1000, abcam).

### Long-term *in vivo* imaging

Healthy 4-5 week-old male Balb/c mice were bought from the SLAC Laboratory Animal Center (Shanghai, China). All animal procedures were approved by the Institutional Animal Ethics Committee of the Institute of Urban Environment, Chinese Academy of Sciences and were carried out in accordance with relevant guidelines and regulations. Five mice were used per time point. Control group Balb/c mice were intravenously injected with 200 μL PBS, whereas the experimental group mice were intravenously injected with 200 μL of ZGO (1 mg/mL, a dose of 10 mg/kg) and imaged at different times (1, 7, 15, 30 and 60 days) after *in situ* irradiation with a 655 nm LED (15 W) for 5 min. The persistent luminescence signals were then collected by an IVIS Lumina II imaging system. The exposure time was set to 60 s.

### Long-term biodistribution

ZGO-injected mice (a dose of 10 mg/kg) were sacrificed at various times (1, 7, 15, 30 and 60 days), and major organs including heart, liver, spleen, lungs, kidney were collected and irradiated with a 655 nm LED for 3 min. The persistent luminescence signals were collected on an IVIS Lumina II imaging system. The exposure time was set to 60 s.

### Blood analysis

ZGO injected mice were sacrificed at various time points (1, 7, 30 and 60 days). The control group mice injected with PBS were also sacrificed at the same time points (n = 5). In order to evaluate the possible effect of persistent luminescence of ZGO *in vivo*, the ZGO pre-irradiated with a 254 nm UV lamp was set as one of the factors. Blood from the mice was drawn through the orbital venous plexus, and approximately a 1 mL portion of blood was collected from each mouse. For a blood chemistry analysis, alkaline phosphatase (ALP), alanine aminotransferase (ALT), aspartate aminotransferase (AST), total bilirubin (TBIL), urea and creatinine (CRE) were measured by the kits according to the guidelines (Nanjing Jiancheng Bioengineering Institute, China). The complete blood panel analysis including red blood cells (RBC), white blood cells (WBC), platelet count (PLT), mean corpuscular volume (MCV), mean corpuscular concentration (MCHC), hemoglobin (HGB), and hematocrit (HCT) were measured by a veterinary hematology analyzer.

### Histological analysis

For histological analysis, major organs including the heart, liver, spleen, lungs and kidneys were collected from mice, fixed in 4% paraformaldehyde, embedded in paraffin, sectioned into slices and stained with hematoxylin/eosin (H&E). The slices were observed with a digital microscope (Olympus, Japan).

### Statistical analyses

All data were expressed as a mean ± SD. Statistical analyses were performed with SPSS 17.0 software (SPSS Inc., Chicago, IL, USA). One-way analysis of variance (ANOVA) followed by the least significant differences (LSD) method after checking the homogeneity of variance was used for comparison of the relevant groups. A p-value of less than 0.05 was considered significant.

### Data availability

All data supporting the conclusions here are available from the authors on reasonable request.

## Electronic supplementary material

Supplementary Information
