# Supplementary material for: Long-term in vivo biodistribution and toxicity study of functionalized near-infrared persistent luminescence nanoparticles
Source: Sci Rep. 2018 Jul 13;8:10595. doi: 10.1038/s41598-018-29019-z (PMC6045641; doi:10.1038/s41598-018-29019-z)
Supplement: Supplementary file 1 — Supplementary Information [file 41598_2018_29019_MOESM1_ESM.pdf]

Supplementary Information

Long-term in vivo biodistribution and toxicity study of functionalized near-infrared persistent luminescence nanoparticles

Xia Sun[1], Junpeng Shi[1], Xiaoyan Fu[3], Yi Yang[1,2], Hongwu Zhang[1*]

1. Key Lab of Urban Pollutant Conversion, Institute of Urban Environment, Chinese Academy of Sciences, Xiamen 361021, China

2. University of Chinese Academy of Sciences, Beijing 100049, China

3. College of Materials Science and Engineering, Xiamen University of Technology, Xiamen 361024, China.

*Correspondence to Hongwu Zhang, Ph.D.

Email: hwzhang@iue.ac.cn

Tel/Fax: +86 592 6190773.

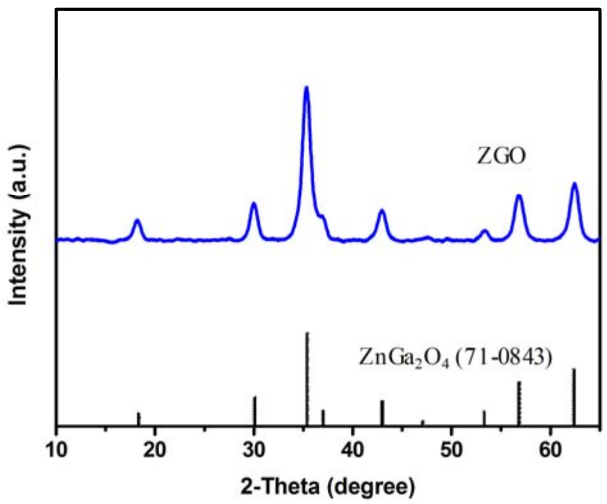

Fig. S1. XRD patterns of ZGO

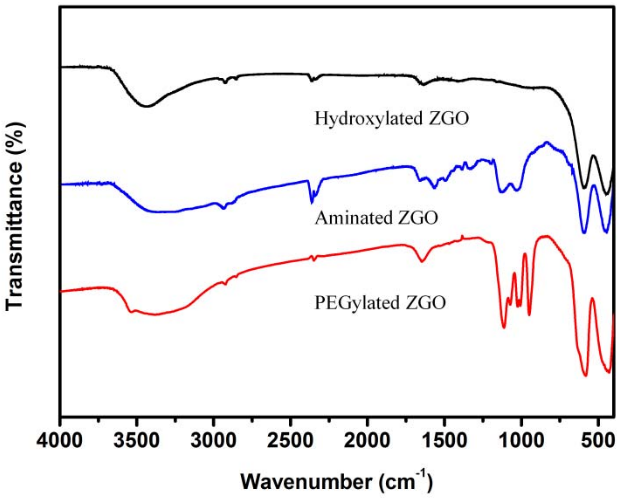

Fig. S2. FTIR spectra of the modification process of ZGO. The FTIR absorption bands at 1030 cm^{-1} and 1129 cm^{-1} (Si-O-Si and C-N stretching vibration), 1567 cm^{-1} (the primary amine groups) confirmed the successful modification of the APTES. The appearance of the absorption bands at 950 and 1110 cm^{-1} (C-O stretching vibration), 1650 cm^{-1} (C=O stretching vibration) and 2850 cm^{-1} and 2920 cm^{-1} (CH_3 stretching vibration) confirmed the successful modification of the PEG to the ZGO surface.

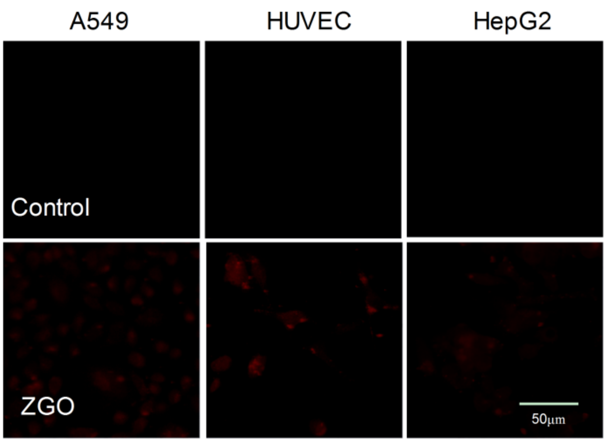

Fig. S3. In vitro cellular uptake of ZGO in three different cell lines.

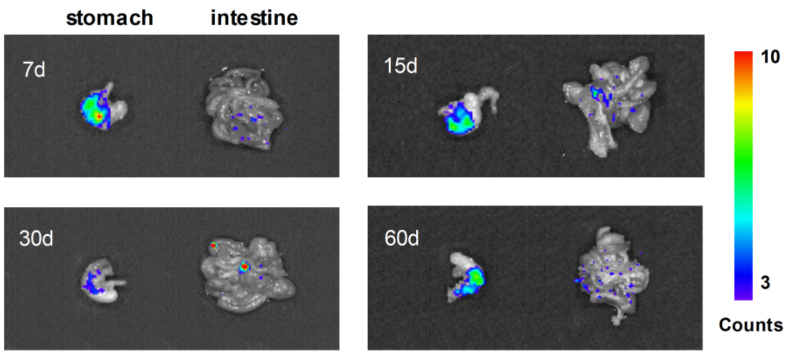

Fig. S4. Persistent luminescence intensities of ZGO from digestive organs (stomach and intestine) at different time points.

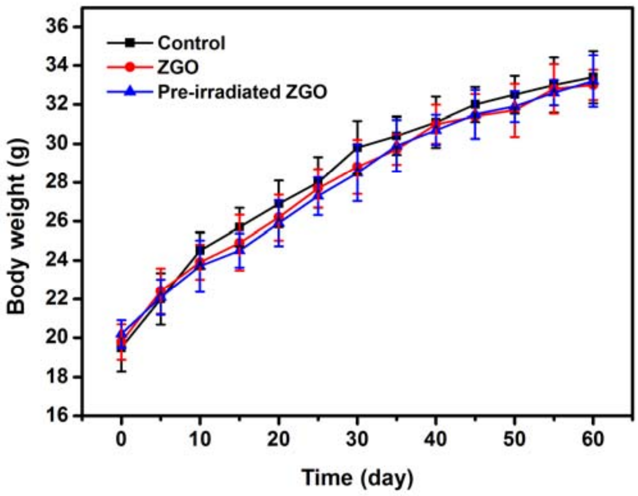

Fig. S5. Body weight changes of Balb/c mice treated with ZGO and pre- irradiated ZGO at different time points. The data represent the mean ± SEM (n =5).

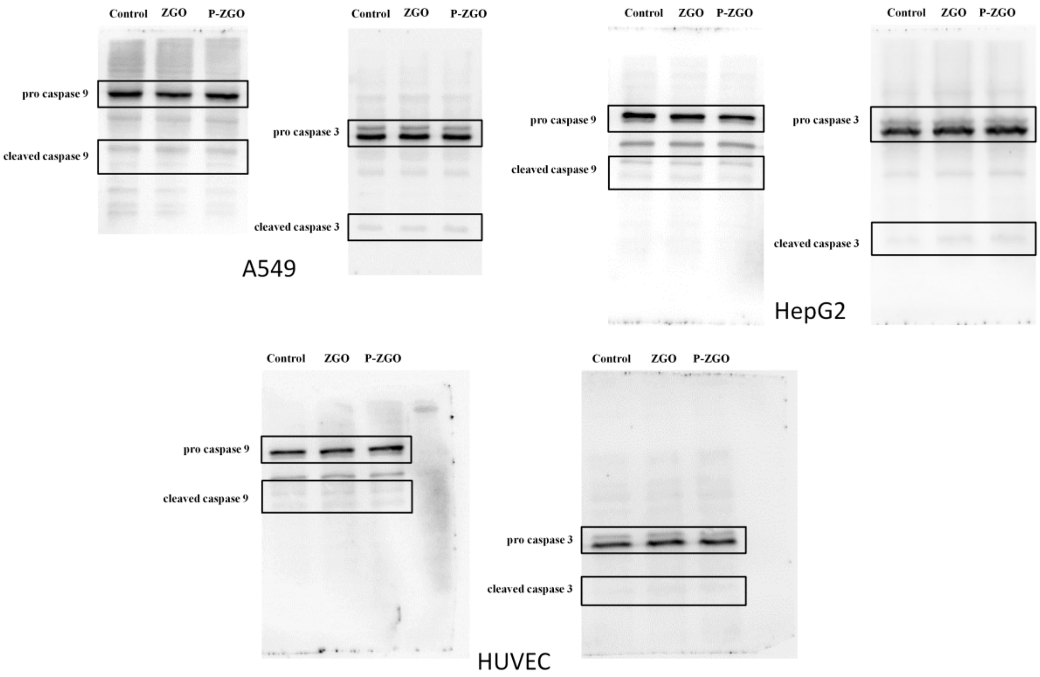

Full-length gels and blots used for Fig.4
